# Histological and immunohistochemical findings in recurrent nevi^☆^

**DOI:** 10.1016/j.abd.2025.501241

**Published:** 2025-11-06

**Authors:** Maisa Aparecida Matico Utsumi Okada, Renata Heck, Renato Marchiori Bakos

**Affiliations:** a Hospital de Dermatologia Sanitária do Paraná, Curitiba, PR, Brazil; b Universidade Federal do Rio Grande do Sul, Porto Alegre, RS, Brazil

**Keywords:** Immunohistochemistry, Nevus, Pigmented, Proto-oncogene proteins B-raf, Recurrence

## Abstract

**Background:**

Recurrent nevi (RN) arise from the incomplete removal of a benign melanocytic lesion. They may present with clinical and dermoscopic characteristics similar to melanomas and are a potential mimic of neoplasia.

**Objective:**

To describe histopathological and immunohistochemical findings of recurrent nevi, including BRAF immunoexpression.

**Methods:**

A cross-sectional study was conducted on 58 recurrent nevi obtained from a previous prospective study. RN were submitted to histopathological and immunohistochemical analysis. The markers HMB-45, Tyrosinase, Ki-67, and BRAF V-600E were used.

**Results:**

A trizonal pattern was observed in 84.5% of cases and is defined by the presence of junctional melanocytic proliferation, scar tissue, and residual dermal nests. Furthermore, architectural asymmetry (56.9%), lentiginous hyperplasia (75.9%), fibrosis (98.4%), lymphocytic infiltrate (96.5%), and melanophages (79.2%) were identified. Dropping off was observed in 1.7%, adnexal involvement in 22.3%, a pagetoid distribution in 30%, and cell atypia in 15%. HMB-45 and tyrosinase were expressed in a gradient and were identified in 98.3% and 91.4%, respectively. Ki-67 was positive in all cases, and the mean proliferative index was 2.83%. BRAF expression was positive in 72.4%.

**Study limitations:**

Limited sample size, evaluation by a single dermatopathologist, targeted immunohistochemical profiling, and the lack of red counterstaining could be potential limitations of the study.

**Conclusions:**

RN are characterized by architectural asymmetry and a trizonal pattern. Nuclear atypia and a pagetoid distribution may be observed. RN cells have a low proliferative index and are positive for HMB-45 and tyrosinase. BRAF expression occurs in most recurrences and is heterogeneous in RN melanocytes.

## Introduction

Recurrent nevi (RN) are pigmented lesions that arise after incomplete removal of a benign melanocytic lesion, usually an acquired melanocytic nevus.^1^ They are more prevalent in females and young adults, with age being a predictor of recurrence.^2^ The incidence of recurrence varies in the literature and is associated with the technique used to remove the primary lesion, with shaving being commonly associated with it.^3^

The genesis of recurrent nevi is not completely understood. Gougerot proposes the seeding of nevus cells during the surgical removal of the nevus.^4^ Schoenfeld and Pinkus suggest that, after partial excision of a nevus, nevus cells present at the dermal-epidermal junction and in the root sheath of the hair follicle would be transferred to the regenerative tissue and form a new junctional nevus.^5^ Cox and Walton propose that new junctional nevus cells are formed after stimuli induced by residual dermal nevus cells.^6^ Imagawa et al. describe junctional proliferative activity derived from hair follicles and eccrine sweat ducts.^7^

The recurrent nevus has clinical and dermoscopic similarities to melanoma.^8^ It presents as a pigmented lesion located over scar tissue, with irregular and asymmetrical contours and coloration that can range from light brown to black. The pigment respects the boundaries of the underlying scar, and the time to recurrence ranges from 6 to 12 months. In contrast, recurrent melanoma may extend beyond the scar boundaries, exhibit continuous growth, and the time to recurrence is generally longer than that of recurrent nevus.^1^

The main dermoscopic features of recurrent nevus include heterogeneous pigmentation, globules, and segmental radial lines. Other possible findings include structureless areas, irregular striations, irregular pigmentary network, and starbursts.^9^, ^10^, ^11^, ^12^, ^13^

In cases where examination of the primary lesion is not available and the diagnosis of melanoma cannot be ruled out, the lesion should be sent for histopathological analysis. Recurrent nevi may present with a trizonal pattern, consisting of junctional melanocytic proliferation over scar tissue and residual dermal nevus cells. Cytological atypia, pagetoid distribution, extension to the adnexa, and dermal inflammation are histological findings described in recurrent nevi and resemble those found in superficial spreading melanoma.^4^, ^14^, ^15^, ^16^, ^17^ This similarity led Kornberg and Ackerman, in 1975, to use the term “pseudomelanoma” for recurrent nevi.^8^ Immunohistochemical examination may be useful in helping to differentiate the two lesions.

Data on the immunohistochemistry of recurrent nevi are scarce and are assumed to be similar to common melanocytic nevi. The objective of this article is to describe the histopathological and immunohistochemical findings of recurrent nevi.

## Methods

A cross-sectional study was conducted with 58 recurrent nevi obtained from a previous prospective study.^2^ In this study, 224 common melanocytic nevi were removed using the shaving technique from 61 patients. All lesions were removed for aesthetic reasons or due to complaints of pruritus or discomfort. The patients were followed for six months, and 58 nevi recurred. Recurrent lesions were excised and submitted to pathological and immunohistochemical examination. The project was approved by the institution's ethics committee, and all patients signed an informed consent form.

Anatomopathological and immunohistochemical examinations were evaluated by an experienced dermatopathologist. Histopathological analysis followed standard techniques and evaluated the following histological findings: architectural symmetry, trizonal pattern, residual nevus cells, lentiginous hyperplasia, pagetoid distribution, confluent melanocytic nests, dropping off, melanophages, fibrosis, inflammation, perineural and periadnexal involvement, mitosis, and nuclear atypia.

The immunohistochemical markers evaluated were: HMB-45, Tyrosinase, Ki-67, and BRAF V-600E. The automated Benchmark ULTRA® platform (Ventana Medical Systems, Tucson, Arizona) was used, and paraffin block samples were cut with a microtome set at 3 μm. Each slide received a positive control, and deparaffinization was performed on the equipment using EZ PREP reagent. Antigen retrieval was performed using Ultra CC1, pH 9.0, at 95 °C for 64 minutes, followed by peroxidase blocking with the UltraView Universal DAB Inhibitor 3% H_2_O_2_ blocking reagent, present in the detection system. The following primary antibodies were used: anti-HMB-45, clone HMB45, dilution 1:100 (Cell Marque®), incubated for 32 minutes at 42 °C; anti-Ki67, clone 30-9 (Roche®), incubated for 16 minutes at 37 °C; and anti-Tyrosinase, clone T311 (Cell Marque®), incubated for 12 minutes at 37 °C. After primary antibody incubation, the reaction was detected with the Ultra View Universal DAB detection kit (multimers), using the Diaminobenzidine (DAB) chromogen present in the kit. The slides were counterstained with Mayer's hematoxylin, differentiated with Bluing reagent (Li_2_CO_3_ + Na_2_CO_3_), and examined after dehydration and mounting.

To process the slides for the BRAF antibody, antigen retrieval was performed at 100 °C, and the antibody used was anti-BRAF, clone VE1 (Roche®), incubated for 20 minutes at 36 °C. The antibody reaction was detected with the Optiview DAB detection system, using the DAB chromogen present in the kit, with the remaining steps being the same as described for the other markers.

Immunohistochemical expression was initially assessed by marker positivity, followed by marker expression assessment through nevus components.

A descriptive study was performed, with the data presented as frequency of occurrence.

## Results

Clinical and epidemiological data on the primary lesions were presented in a previously published study.^2^

The histological findings are shown in Table 1.

**Table 1:** Histological characteristics of recurrent nevi.

Histological findings	Number of lesions (%)
Trizonal pattern	49/58 (84.5%)
Architectural asymmetry	33/58 (56.9%)
Lentiginous hyperplasia	44/58 (75.9%)
Fibrosis	57/58 (98.3)
Inflammatory infiltrate	56/58 (96.6%)
Dropping off	1/58 (1.7%)
Adnexal involvement	13/58 (22.3%)
Melanophages	46/58 (79.2%)
Pagetoid distribution	17/58 (29.3%)
Cell atypia	9/58 (15.5%)
Mitoses	0/58 (0%)

**Fig. 1:**
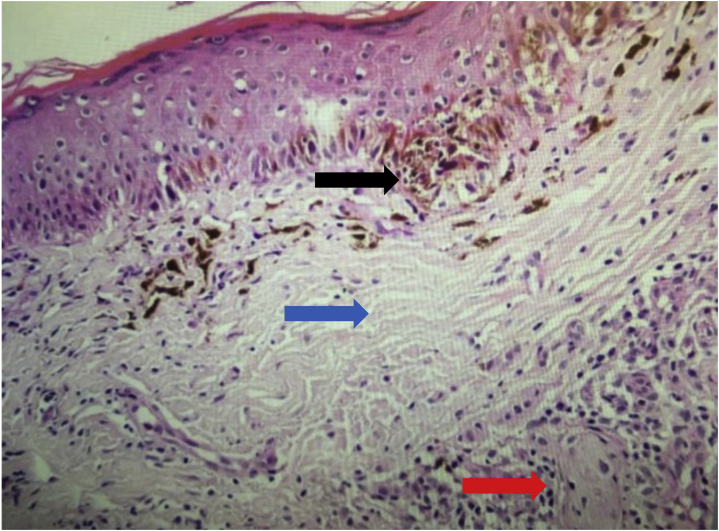
Anatomopathological image of recurrent melanocytic nevus showing a trizonal pattern: lentiginous junctional component (black arrow), cicatricial dermal fibrosis (blue arrow), nests of nevus cells (red arrow; Hematoxylin & eosin, ×200).

The trizonal pattern was present in 84.5% of recurrent nevi (Fig. 1) and architectural asymmetry in 56.9%, characterized by asymmetry in the contours, size, and shape of nevus cell nests, distribution in the intraepithelial and dermal components, and inflammatory infiltrate.

Mild to moderate lentiginous hyperplasia was identified in 75.9%, fibrosis in 98.4%, and lymphocytic inflammatory infiltrate in 96.5%, graded as mild and located in the superficial dermis in 65.5%. Adnexal involvement was identified in 22.3%, melanophages in 79.2%, and dropping off in 1.7%, defined as detachment of melanocytes from the epidermis into the scar tissue that separates the epidermal from the dermal component in the recurrent nevus.^15^

A pagetoid distribution was observed in 29.3% of the cases, which was classified as mild to moderate. Cell atypia was found in 15%, with no cases of marked atypia. No mitoses were observed in the sample.

**Fig. 2:**
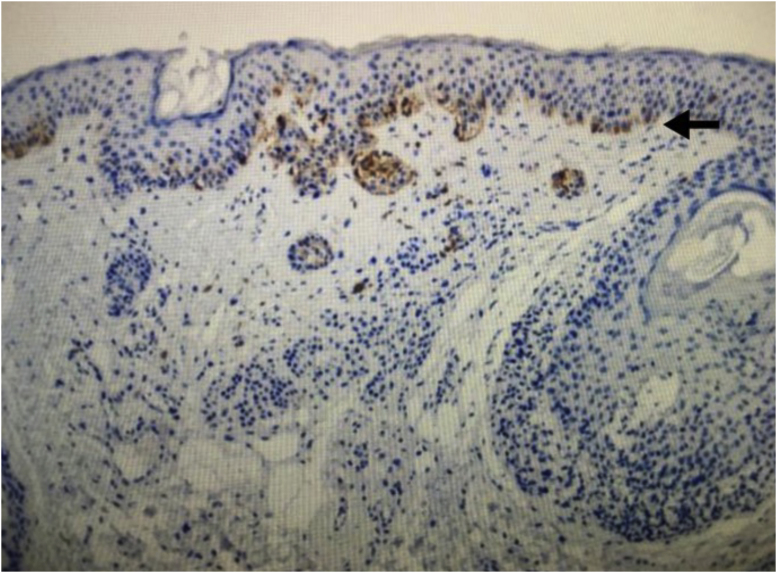
Immunohistochemical analysis image of recurrent melanocytic nevus showing a gradient pattern: strongly positive expression in the junctional component (black arrow) and gradually decreasing expression in the deep components. (HMB-45 immunostaining ×100).

**Fig. 3:**
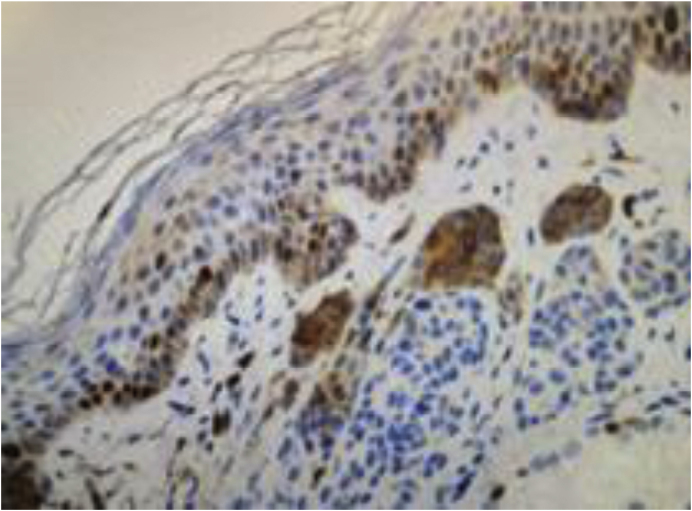
Image of immunohistochemical analysis of recurrent melanocytic nevus showing staining for the Ki-67 antibody. (Ki-67 immunostaining ×200).

**Fig. 4:**
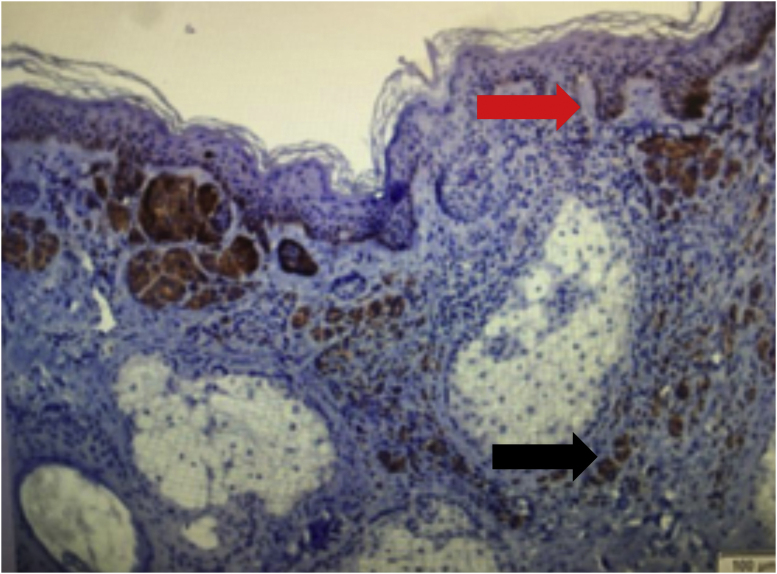
Image of immunohistochemical analysis of recurrent melanocytic nevus showing the expression of the BRAF mutation in the junctional (red arrow) and dermal (black arrow) components (BRAF immunostaining ×100).

Regarding the immunohistochemical markers, shown in Table 2, HMB-45 was positive in 98.3% (57/58) and was expressed in a gradient, being strongly positive in the junctional component and with decreased staining in the deeper layers of the dermis (Fig. 2). Tyrosinase showed the same expression pattern as HMB-45 and was positive in 91.4% (53/58) of the cases. Ki-67 was positive in all recurrences and showed a staining index ranging from 1% to 8%, with a mean of 2.83% (Fig. 3). All lesions showed staining in the junctional component and only one lesion showed positivity in the dermal nests. BRAF positivity in nevi was 72.4% (42/58; Table 3). BRAF expression was observed in the junctional portion of seven lesions, in the dermal component of 28, and in both in seven lesions (Fig. 4).

**Table 2:** Immunohistochemical characteristics of recurrent nevi.

Immunohistochemical antibody	Number of lesions with positive expression
HMB-45	57/58 (98.3%)
Tyrosinase	53/58 (91.4%)
Ki-67	58/58 (100%)
BRAF V600E	42/58 (72.4%)

**Table 3:** BRAF expression in recurrent nevi.

Local of BRAF expression	*n* (%)
Junctional	7 (16.7%)
Compound	7 (16.7%)
Dermal	28 (66.7%)

## Discussion

This study evaluated the histopathological and immunohistochemical characteristics of RN. The markers HMB-45 and tyrosinase were positive in most lesions and were expressed in a gradient. Recurrences showed a low proliferative index, as assessed by Ki-67, and immunohistochemical staining of the BRAF mutation occurred in 72.4%. Clinical and epidemiological data of recurrent nevi have been described in a previous study.^2^

Few studies have explored the histopathological findings of RN. In a sample of 175 recurrent nevi, Park et al. classified them into four histological types: lentiginous, junctional, compound, and intradermal. Compound nevi show a trizonal appearance, consisting of junctional melanocytic proliferation, scar tissue, and residual dermal nests, and constituted 52% of their sample.^15^ The present study identified a trizonal pattern in 84.5%. Interestingly, in the present study, the majority of primary nevi (84.7%) were histologically classified as intradermal nevi and only 15.3% as compound nevi.^2^ Park reports exaggerated junctional activity in their sample and suggests that melanocytes located at the dermal-epidermal interface could give rise to recurrences, whether located in the eccrine glands, hair follicles, or adjacent skin.^15^ In the present sample (unpublished data), the margins were compromised in all primary lesions submitted to the shaving technique. These findings may suggest that perilesional melanocytes could repopulate the epidermis and that junctional activity in the recurrent nevus may be responsible for its formation. Botella-Estrada et al.^18^ evaluated pigmented lesions in scar tissue after excision of melanoma and non-melanocytic tumors. The authors found melanocytic hyperplasia in both scenarios and suggest that scar tissue acts by inducing melanocytes from the overlying epidermis. Although the authors state that this scenario is distinct from recurrent nevi, given the partial removal of the primary lesion in the latter case, one cannot exclude the effect of scar tissue on epidermal melanocytes. Further studies are needed to clarify the pathophysiology of recurrent nevi.

Regarding architectural symmetry, the present study observed asymmetry in 56.9%. In turn, Hoang et al. identified asymmetry in 46.6% of recurrent nevi. Adnexal involvement was identified in 22.3% of recurrent nevi. King et al. reported melanocyte extension into adnexal areas in 6% of cases, similar to Hoang's study. This difference can be explained by the location of the precursor nevi. The back was the most frequent anatomical site in the study by King (57%) and Hoang (33.3%).^14^, ^17^ In the present study, the face was the site of highest frequency of recurrences (45.8%), followed by the back (22%).^2^

Park identified a marked pagetoid distribution in 3%, mild atypia in 30%, and moderate atypia in 12%.^15^ King et al. reported a pagetoid distribution in 6% and cell atypia in 26%, defined as the presence of hyperchromasia and enlarged nuclei. The authors observed that most recurrent nevi are easily recognizable histologically. However, RN that show flattening of the dermal-epidermal junction combined with a pagetoid distribution and adnexal involvement may be histologically indistinguishable from melanomas in late regression.^14^

Few studies have evaluated the immunohistochemical characteristics of recurrent nevi. Hoang et al. evaluated the expression profile of S-100, MART-1, HMB-45, tyrosinase, and Ki-67 in 15 recurrent nevi. The authors found that HMB-45 and tyrosinase were positive in 90% of recurrent nevi, and the staining followed a gradient pattern, similar to that of acquired melanocytic nevi and in agreement with the present study.^17^

HMB-45 is a monoclonal antibody targeting the glycoprotein gp100, a component of the premelanosome, and it marks both activated and immature melanocytes. In benign lesions, the immunohistochemical staining pattern is similar to that of melanocyte maturation, i.e., a more strongly positive junctional component and progressively less staining as the lesion deepens into the dermis. Tyrosinase, in turn, is a cytoplasmic enzyme involved in melanin synthesis and is also expressed in a gradient in benign melanocytic lesions. The use of these markers can be a useful tool in differentiating recurrent nevi from melanoma, which shows diffuse staining.^19^, ^20^

When evaluating Ki-67 in recurrent nevi, it was observed that all were positive for this marker, with a mean proliferative index of 2.83%. All lesions showed positivity in their junctional component, except for one that showed positivity in dermal nests. The study by Hoang also identified low proliferative indices in recurrent nevi, both in the intraepidermal and dermal components.^17^ The present finding is consistent with the data on Ki-67 in melanocytic nevi.^21^ In benign melanocytic lesions, the proliferation index is generally below 5%, and staining predominates in the dermal-epidermal junction or superficial dermis. In melanoma, up to 50% of cells are stained, with diffuse and heterogeneous staining.^21^, ^22^, ^23^

The use of immunohistochemical markers such as HMB-45 or tyrosinase, combined with Ki-67, can help differentiate recurrent nevi from melanoma. Uguen et al. used a panel of markers consisting of Ki-67, p16, and HMB-45 to differentiate melanocytic nevi from melanoma. Ki-67 alone was the most effective marker for diagnosing melanoma, but it fails in cases of poorly proliferative melanomas.^24^ Further studies evaluating the immunohistochemical characteristics of recurrent nevi are needed to define which markers are most effective in differentiating these lesions from melanoma.

The BRAF oncogene mutation was initially described in melanoma, and subsequent studies identified the mutation in melanocytic nevi. The high prevalence of the mutation in acquired melanocytic nevi suggests that the BRAF mutation is an initial event in melanocyte proliferation and nevus formation.^25^ In melanoma, the BRAF mutation is associated with young age and intermittent sun exposure. In acquired melanocytic nevi, this mutation is associated with a globular dermoscopic pattern and growing nevi.^26^ These findings may suggest that the BRAF mutation may be involved in melanocyte proliferation and the onset of recurrences. To the best of the authors’ knowledge, this study is the first to describe the immunohistochemical expression of the BRAF mutation in recurrent nevi.

Investigating BRAF expression in recurrent nevi may help understand the phenomenon of recurrence, which has not yet been fully elucidated. In the present study, the mutation was expressed in 72.4% of recurrences, which is similar to the rates found in acquired melanocytic nevi in ​​general.^27^ It was also observed that in most recurrences (66.7%), BRAF immunoexpression was expressed only in the dermal component. This finding may suggest that recurrences may also originate from the proliferation of residual dermal melanocytes. In acquired melanocytic nevi, mutation expression occurs homogeneously throughout the nevus components.^26^, ^28^ Furthermore, most recurrent nevi show heterogeneous BRAF immunoexpression, with isolated staining in the dermal or junctional component. These findings, combined with the observation of increased junctional activity in the recurrent nevus, may suggest that recurrences are polyclonal in origin and arise from cells that may or may not harbor the mutation.

The authors believe this study shows consistent data regarding the histopathological and immunohistochemical findings of recurrent nevi. However, it may have potential limitations, including: a small sample of recurrent nevi, analysis by only one dermatopathologist, use of a limited immunohistochemical panel to characterize recurrent nevi, and the lack of magenta counterstaining for BRAF expression analysis.

## Conclusion

Recurrent nevi are primarily characterized by architectural asymmetry and a trizonal pattern. Atypical findings such as nuclear atypia and pagetoid distribution may be present in recurrent nevi, which may mimic melanoma both clinically and histologically. Immunohistochemistry can be of great assistance in their differentiation. In recurrent nevi, BRAF expression is present in most lesions and seems to occur heterogeneously among melanocytes. Further studies are needed to clarify the recurrence process and investigate its association with BRAF mutations.

## ORCID IDs

Maisa Aparecida Matico Utsumi Okada: 0000-0002-1497-010X

Renata Heck: 0000-0003-2352-3915

Renato Marchiori Bakos: 0000-0002-8114-246X

## Authors’ contributions

Maisa Aparecida Matico Utsumi Okada: Data collection, or analysis and interpretation of data; drafting and editing of the manuscript or critical review of important intellectual content; collection, analysis and interpretation of data; critical review of the literature; approval of the final version of the manuscript.

Renata Heck: Data collection, or analysis and interpretation of data; drafting and editing of the manuscript or critical review of important intellectual content; collection, analysis and interpretation of data; critical review of the literature; approval of the final version of the manuscript.

Renato Marchiori Bakos: Design and planning of the study; effective participation in research orientation; approval of the final version of the manuscript.

## Financial support

This work received financial support from *F**undação de Apoio à Dermatologia* (FUNADERM).

## Availability of research data

The entire dataset supporting the results of this study was published in the article itself.

## Declaration of competing interest

None declared.

## References

[1] Fox J.C., Reed J.A., Shea C.R. (2011). The recurrent nevus phenomenon: a history of challenge, controversy, and discovery. Arch Pathol Lab Med.

[2] Heck R., Ferrari T., Cartell A., Bakos R.M. (2019). Clinical and dermoscopic (in vivo and ex vivo) predictors of recurrent nevi. Eur J Dermatol.

[3] Goodson A.G., Florell S.R., Boucher K.M., Grossman D. (2010). Low rates of clinical recurrence after biopsy of benign to moderately dysplastic melanocytic nevi. J Am Acad Dermatol.

[4] Gougerot H. (1947). Récidive des naevi, après destruction, dans le même type, dans la même forme, dans la même étendue. Monde Méd.

[5] Schoenfeld R.J., Pinkus H. (1958). The recurrence of nevi after incomplete removal. AMA Arch Derm.

[6] Walton R.G., Sage R.D., Farber E.M. (1957). Electrodesiccation of pigmented nevi; biopsy studies: a preliminary report. AMA Arch Derm.

[7] Imagawa I., Endo M., Morishima T. (1976). Mechanism of recurrence of pigmented nevi following dermabrasion. Acta Derm Venereol.

[8] Kornberg R., Ackerman A. (1975). Pseudomelanoma: recurrent melanocytic nevus following partial surgical removal. Arch Dermatol.

[9] Botella-Estrada R., Nagore E., Sopena J., Cremades A., Alfaro A., Sanmartín O. (2006). Clinical, dermoscopy and histological correlation study of melanotic pigmentations in excision scars of melanocytic tumours. Br J Dermatol.

[10] Blum A., Hofmann-Wellenhof R., Marghoob A.A., Argenziano G., Cabo H., Carrera C. (2014). Recurrent melanocytic nevi and melanomas in dermoscopy: results of a multicenter study of the international dermoscopy society. JAMA Dermatol.

[11] Tschandl P. (2013). Recurrent nevi: report of three cases with dermatoscopic-dermatopathologic correlation. Dermatol Pract Concept.

[12] Yoshida Y., Yamada N., Adachi K., Tanaka M., Yamamoto O. (2008). Traumatized recurrent melanocytic naevus with typical starburst pattern on dermoscopy. Acta Derm Venereol..

[13] Moscarella E., Argenziano G., Lallas A., Longo C., Al Jalbout S., Zalaudek I. (2013). Pigmentation in a scar: use of dermoscopy in the management decision. J Am Acad Dermatol.

[14] King R., Hayzen B.A., Page R.N., Googe P.B., Zeagler D., Mihm M.C. (2009). Recurrent nevus phenomenon: a clinicopathologic study of 357 cases and histologic comparison with melanoma with regression. Mod Pathol.

[15] Park H.K., Leonard D.D., Arrington J.H., Lund H.Z. (1987). Recurrent melanocytic nevi: clinical and histologic review of 175 cases. J Am Acad Dermatol.

[16] Vilain R.E., McCarthy S.W., Scolyer R.A. (2016). The regenerating naevus. Pathology.

[17] Hoang M.P., Prieto V.G., Burchette J.L., Shea C.R. (2001). Recurrent melanocytic nevus: a histologic and immunohistochemical evaluation. J Cutan Pathol.

[18] Botella-Estrada R., Sanmartín O., Sevila A., Escudero A., Guillén C. (1999). Melanotic pigmentation in excision scars of melanocytic and non-melanocytic skin tumors. J Cutan Pathol.

[19] Fuertes L., Santonja C., Kutzner H., Requena L. (2013). Immunohistochemistry in dermatopathology: a review of the most commonly used antibodies (part II). Actas Dermosifiliogr.

[20] Ordóñez N.G. (2014). Value of melanocytic-associated immunohistochemical markers in the diagnosis of malignant melanoma: a review and update. Hum Pathol.

[21] Vyas N.S., Charifa A., Desman G.T., Goldberg M., Singh R., Phelps R.G. (2019). Observational study examining the diagnostic practice of Ki67 staining for melanocytic lesions. Am J Dermatopathol.

[22] Nasr M.R., El-Zammar O. (2008). Comparison of pHH3, Ki-67, and survivin immunoreactivity in benign and malignant melanocytic lesions. Am J Dermatopathol.

[23] Prieto V.G., Shea C.R. (2008). Use of immunohistochemistry in melanocytic lesions. J Cutan Pathol.

[24] Uguen A., Talagas M., Costa S., Duigou S., Bouvier S., De Braekeleer M. (2015). A p16-Ki-67-HMB45 immunohistochemistry scoring system as an ancillary diagnostic tool in the diagnosis of melanoma. Diagn Pathol.

[25] Gill M., Celebi J.T. (2005). B-RAF and melanocytic neoplasia. J Am Acad Dermatol.

[26] Marchetti M.A., Kiuru M.H., Busam K.J., Marghoob A.A., Scope A., Dusza S.W. (2014). Melanocytic naevi with globular and reticular dermoscopic patterns display distinct BRAF V600E expression profiles and histopathological patterns. Br J Dermatol.

[27] Damsky W.E., Bosenberg M. (2017). Melanocytic nevi and melanoma: unraveling a complex relationship. Oncogene.

[28] Yeh I. (2020). New and evolving concepts of melanocytic nevi and melanocytomas. Mod Pathol.

